# A Simplified Working Classification for Planning and Management of Facial Fractures

**DOI:** 10.1055/s-0045-1802344

**Published:** 2025-01-28

**Authors:** Mukesh K. Sharma, Rahul Saini, Bulli B. Boyidi, Vaddi S. Babu

**Affiliations:** 1 Department of Burns and Plastic Surgery, Atal Bihari Vajpayee Institute of Medical Sciences and Dr. Ram Manohar Lohia Hospital, New Delhi, India

**Keywords:** facial fractures, buttresses, Le Fort, open reduction and internal fixation, RML classification

## Abstract

Facial fractures are commonly encountered by plastic and maxillofacial surgeons. Although very diversified in nature, their treatment planning requires a thorough knowledge of the facial anatomy and advanced treatment modalities. With the advent of three-dimensional computed tomography, it has become a lot easier to diagnose and treat them accordingly. It is important to categorize facial fractures for an effective liaison between the radiologists, surgeons, and medical staff involved in their management. Various classification schemes have been made to classify them, but they are cumbersome to remember and communicate among treating doctors. We present a new yet simple facial fracture classification that is based on the facial buttresses involved. This helps in better and uniform management of fracture patterns and also anticipates future complications that may arise from such fractures, if any.

## Introduction

Facial fractures are increasing in number due to the ever increasing rise of road traffic accidents (RTAs) and physical assault worldwide. 1 Interpretation of three-dimensional (3D) images of a facial computed tomography (CT) scan is used as a guide by the operating surgeons to reduce the fracture fragments and restore their alignment, both functionally and aesthetically. There is a lack of clarity among radiologists, surgeons, and technical personnel regarding various kinds of facial fractures involving multiple bones and the issues that need to be resolved during maxillofacial surgery. Simply listing the fractured bones does not emphasize the surgical plan to be made. To facilitate efficient management, CT reports should have anatomic descriptors and classification methods that can be easily communicated and understood among surgeons, radiologists, and medical staff. 1 Hence, we propose a working classification of facial fractures based on 3D-CT images.

We described the fractures in relation to the facial buttresses that need fixation. The vertical buttresses are denoted with “V” and the horizontal buttresses with “H.” Prefixes “R” and “L” are added to the above combined fracture pattern to mark the right and left sides of the face, respectively. The suffix “#” is added to denote fracture.

**Fig. 1:**
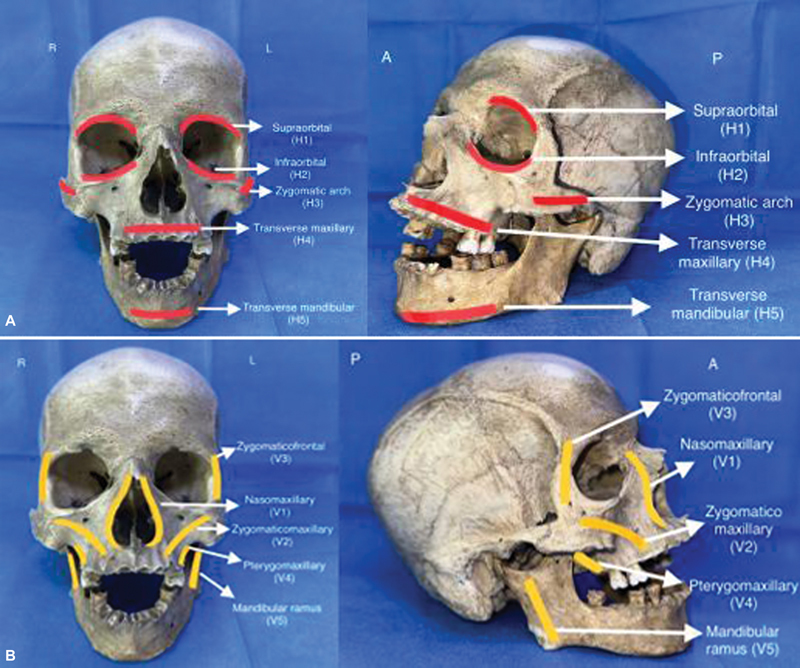
( **A** ) Depiction of horizontal buttresses in frontal view and left oblique view; ( **B** ) depiction of vertical buttresses in frontal view and right lateral view (R: right, L: left, A: anterior, P: posterior, H: horizontal, V: vertical).

Horizontal buttresses are labeled with numerical subscripts as follows, from superior to inferior in order ( Fig. 1A ):

H _1_ : Supraorbital
H _2_ : Infraorbital
H _3_ : Zygomatic arch
H _4_ : Transverse maxillary
H _5_ : Transverse mandibular

Vertical buttresses are labeled with numerical subscripts as follows, from medial to lateral in order ( Fig. 1B ):

V _1_ : Nasomaxillary
V _2_ : Zygomaticomaxillary
V _3_ : Zygomaticofrontal
V _4_ : Pterygomaxillary
V _5_ : Mandibular ramus

Sample diagnosis after following the above rules of our classification: R-H _12345_ V _12345_ / L-H _12345_ V _12345_ #.

### Case Example

**Fig. 2:**
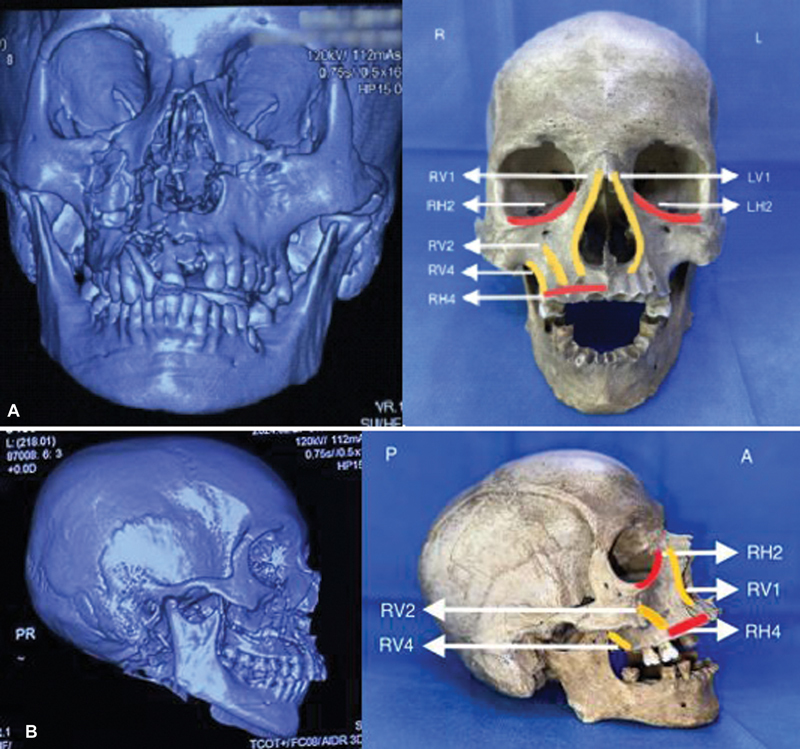
( **A** ) Frontal view and ( **B** ) right lateral view images of preoperative 3D-CT scan images of a facial fracture case (right side) corresponding to the involved fractured buttresses as depicted on a specimen skull (left side). Buttresses involved: H _2_ —infraorbital, H _4_ —transverse maxillary, V _1_ —nasomaxillary, V _2_ —zygomaticomaxillary, V _4_ —pterygomaxillary (R: right, L: left, A: anterior, P: posterior, H: horizontal, V: vertical). 3D-CT, three-dimensional computed tomography.

**Fig. 3:**
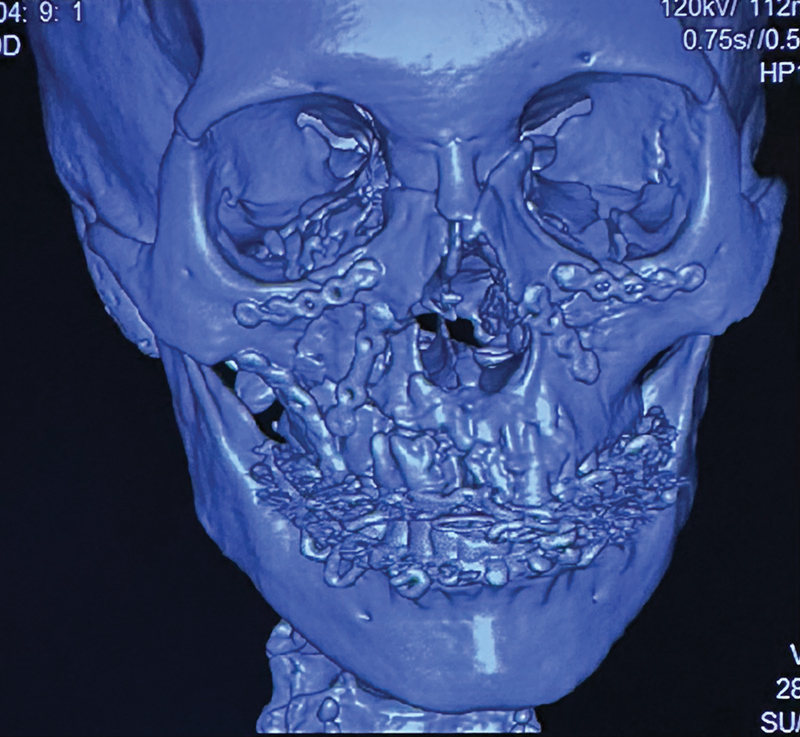
Frontal view of the postoperative 3D-CT face image showing fixed buttresses with miniplates. 3D-CT, three-dimensional computed tomography.

A patient sustained fractures of right-sided nasomaxillary, zygomaticomaxillary, pterygomaxillary, infraorbital, and transverse maxillary buttresses, along with fractures of nasomaxillary and infraorbital buttresses on the left side ( Fig. 2A, B ). According to our classification system, the case diagnosis is “R-H _24_ V _124_ /L-H _2_ V _1_ #.” Patient underwent open reduction and internal fixation of the involved fractured buttresses under general anesthesia. Follow-up postoperative 3D-CT face images of the patient were taken that confirmed various buttresses that were fixed using miniplates and screws ( Fig. 3 ).

## Discussion

The human facial skeleton is constituted by five paired and four unpaired bones. As it can be inconvenient to describe facial fractures according to the facial bones involved, the bony structure is simplified into horizontal and vertical buttresses. Buttresses are areas of thick bones in the facial skeleton that maintain facial width, projection, and height. Nasomaxillary, zygomaticomaxillary, zygomaticofrontal, pterygomaxillary, and the mandibular ramus are the vertical buttresses. Horizontal buttresses include the frontal bar, inferior orbital rim, zygomatic arch, transverse maxillary, and transverse mandibular. 2 The surgical fixation of facial fractures is directed at the fixation of these buttresses, as they have good bone stock.

Although a clinical examination can rule out facial fractures in a subset of patients with low impact energy, 3 classification of facial fractures cannot be done by physical examination alone due to facial swelling, obtundation and distracting injuries. 4 Thus, for planning a surgical approach, imaging is critical for surgeons to understand which buttresses are involved. 5 6 Their diagnosis and management have become much more refined because of the better availability of hardware and multidimensional CT-scan as the imaging standard. 5

Each individual fracture pattern is associated with a particular functional and aesthetic outcome. Hence, there is significance in knowing typical patterns and classifications of facial fractures. 7 Various sequences for multiple facial fracture repair, such as bottom to top, top to bottom, lateral to medial and medial to lateral approaches, have been described. The AO Craniomaxillofacial classification of cranio-maxillofacial fractures in adults involves three levels of precision to describe these injuries. Level 1 conveys the presence or absence of fractures in four basic anatomical units, i.e., the mandible, mid-face, skull base, and cranial vault. Level 2 delineates the site of fractures in detail within specific areas of the mandible, central and lateral mid-face, internal orbit, endocranial and exocranial skull base, and cranial vault. Level 3 gives the finest detail about the location of injury, including the morphology (fragmentation, displacement, and bone defects) within the previously described subsites. 8

Another classification, described by René Le Fort in 1901, describes various patterns of mid-face fractures, all including fractures of pterygoid plates. 9 Each Le fort fracture type has a fracture component unique to it. The anterolateral margin of the nasal fossa in Le fort 1, the inferior orbital rim in type 2, and the zygomatic arch fracture in type 3 are the components unique to each type. 10 Le fort patterns have certain disadvantages. First, they are based on low-velocity trauma. But nowadays, mostly high-velocity trauma occurs in RTA that leads to different mid-face fracture patterns. 11 Second, due to the widespread availability of good osteosynthetic hardware, the current focus is on the alignment of individual buttresses to restore the required facial alignment. Third, it does not adequately reflect the complexity of the individual components of the mid-facial region. Lastly, the Markowitz and Manson classification, which is used for naso-orbito-ethmoid fractures, is based on the extent of medial canthal tendon involvement. 12

The Dr RML hospital classification system for maxillofacial fractures, as proposed by us, has the benefit of being simple, quick, and reproducible. It is based on the fact that if the involved facial buttresses are fixed, the reduction of facial fractures occurs. 2 It provides better interpersonal communication amongst consultant surgeons and surgical residents, which helps in quick assessment of surgical planning and hardware requirements. On the other hand, this classification does not include fractures of mandibular condyle, frontal bone, naso-orbito-ethmoid, orbital floor, and hard palate. 10 Hence, they can be separately included along with our proposed Dr RML classification system to maintain uniform communication.

## Conclusion

**Fig. 4:**
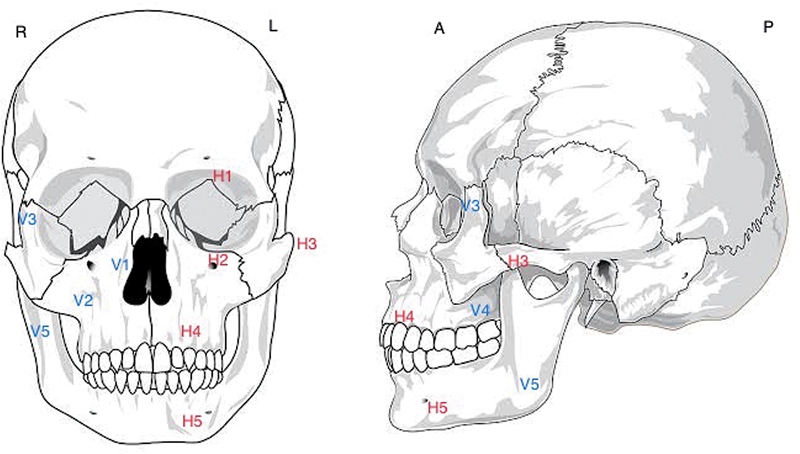
Schematic diagram of the skull depicting facial buttresses as per the RML classification system (R-H _12345_ V _12345_ /L-H _12345_ V _12345_ #).

Universal numbering of various facial buttresses is one of the methods of classification of facial fractures, as proposed in our department and named Dr. RML Hospital classification system ( Fig. 4 ). The use of common terminologies helps in easy interpretation and communication among surgeons, radiologists, and other team members for functional and aesthetic restoration of maxillofacial fractures. Also, our classification can help in easy and rapid assessment by doctors who deal with facial trauma patients in emergencies every day.
